# Amyloidosis of lacrimal gland

**DOI:** 10.4103/0301-4738.57160

**Published:** 2009

**Authors:** Venkatesh C Prabhakaran, Kalpana Babu, Anitha Mahadevan, Sowmya Raveendra Murthy

**Affiliations:** 1 Department of Orbit and Oculoplastic Surgery, Prabha Eye Clinic and Vittala International Institute of Ophthalmology, NIMHANS, Bangalore, India; 2 Department of Ophthalmic Pathology, Prabha Eye Clinic and Vittala International Institute of Ophthalmology, NIMHANS, Bangalore, India; 3 Department of Neuropathology, Prabha Eye Clinic and Vittala International Institute of Ophthalmology, NIMHANS, Bangalore, India

**Keywords:** Amyloidosis, lacrimal gland, localized

## Abstract

Primary localized amyloidosis of lacrimal gland is a rare occurrence. This report describes a female patient with isolated amyloidosis of the lacrimal gland. A 45-year-old Indian woman presented with a swelling over the left lacrimal gland region. Computed tomography showed uniform enlargement of the lacrimal gland. A lacrimal gland biopsy revealed amyloidosis. No systemic involvement was detected on further investigation. To our knowledge, this is the first report of lacrimal gland amyloidosis from India and our report also highlights the importance of lacrimal gland biopsy in diagnosing lacrimal gland masses.

Amyloidosis is a disorder of protein metabolism characterized by extracellular deposition of abnormal protein fibrils.[1,2] It can occur in isolation affecting the ocular and periocular structures or as part of a systemic disease causing organ damage and serious morbidity.[1,2] Primary localized amyloidosis of the orbit is a rare condition with approximately 24 cases reported in literature. Amyloidosis limited to the lacrimal gland is even rarer with only 7 cases reported in literature.[1,2,4,5,6] We report a patient with isolated lacrimal gland amyloidosis.

## Case Report

**Figure 1:**
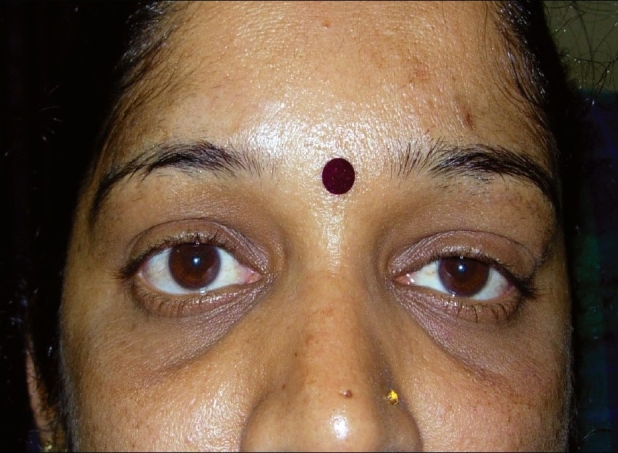
Clinical photograph showing left ptosis and inferior globe displacement

A 45-year-old Indian woman presented with a slowly progressive swelling of the left upper eyelid of three months duration. This was associated with discomfort but was not painful. On examination, the best-corrected visual acuity was 20/20 in both eyes. Left eye examination showed lateral ptosis with S-shaped deformity of the lid [Fig. 1]. A firm, non-tender mass was palpated in the supero-temporal orbit. Hertel's exophthalmometry showed a 2 mm proptosis of the left eye with 2 mm of inferior displacement. The extraocular movements were restricted in levo-elevation, but diplopia could not be elicited. The anterior segment and fundus examinations were normal. The right eye examination was unremarkable.

**Figure 2:**
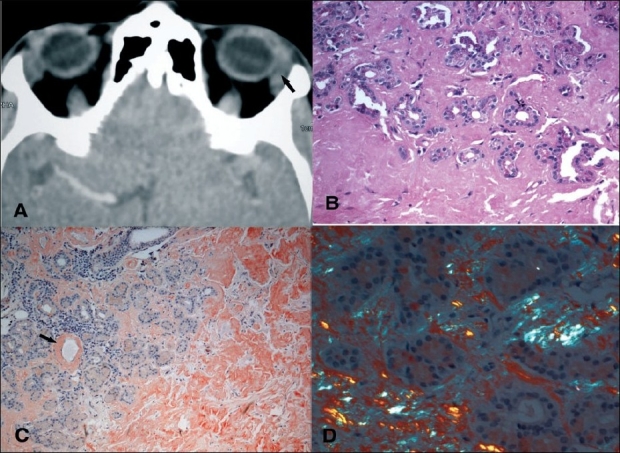
(A) Computed tomography scan (axial view) showing welldefined enlargement of left lacrimal gland (arrow), (B) Photomicrograph of lacrimal gland biopsy illustrating acini infiltrated with amorphous eosinophilic material which has completely replaced the acini in certain areas (H & E, ×100), (C) Section stained with Congo red showing brick-red staining of the amorphous material consistent with amyloid replacing the entire lobule on the right side and deposited in the perivascular region (arrow) (Congo red stain, original magnification ×200), (D) Section stained with Congo red and viewed under polarized light illustrates the apple-green birefringence characteristic of amyloidosis

Computed tomography (CT) scan showed a well-defined, homogenous enlargement of the left lacrimal gland with no indentation of the globe [Fig. 2A]. No calcification or bony erosion was noted. Routine blood tests including total and differential counts and erythrocyte sedimentation rate were within normal limits. Tests for rheumatoid factor and anti-nuclear antibody were also normal. Following discussion with the patient, an incisional biopsy of the left lacrimal gland was performed via a lid crease approach. Peroperatively, the lacrimal gland was found to be enlarged, with a firm consistency and a yellowish tinge.

Histopathological examination showed lobules of lacrimal gland acini that were extensively replaced by amorphous, eosinophillic hyaline deposits [Fig. 2B]. In some areas, these deposits were noted within the acini. On Congo red staining, these deposits stained a brick-red color [Fig. 2C] and revealed green birefringence when observed by polarizing microscope [Fig. 2D]. Immunohistochemistry for lambda and kappa light chains failed to reveal monoclonality. Serum protein electrophoresis did not show any abnormal bands and urine protein analysis was negative. The patient was referred to an immunologist for systemic examination, which did not reveal any evidence of systemic disease. A diagnosis of isolated lacrimal gland amyloidosis was therefore made. The patient was offered the option of further debulking, but she refused and opted to be under observation.

## Discussion

To our knowledge, this is the first case report of lacrimal gland amyloidosis from India.

Amyloidosis is not a common disease and its deposition can be localized (to one organ or body site) or systemic.[1,2] Usual sites of ocular deposits include eyelids, conjunctiva, and cornea. Unlike amyloidosis of skin or eyelids, which is usually associated with generalized amyloidosis, orbital amyloidosis is almost always a benign localized disease.[2,3]

We describe a female patient with unilateral lacrimal gland amyloidosis. She was middle-aged with no associated systemic disease. Orbital amyloidosis is uncommon with only two large case series,[1,4] and isolated lacrimal gland amyloidosis appears to be an exceptionally rare condition with very few cases previously reported.[1,2,4–6] It has been stated that unilateral lacrimal gland involvement is characteristic of isolated amyloidosis whereas bilateral involvement is seen with systemic disease.[1,4]

However, one case of isolated bilateral lacrimal gland involvement has been reported by Conlon *et al*.[5]

The common presenting features of orbital amyloidosis include ptosis, proptosis and globe displacement with supero-temporal orbital mass. Computed tomography scan is not diagnostic but is important in localizing the involved orbital structures. Lacrimal gland involvement is seen as a homogenous soft tissue mass with slightly higher density than the brain. The lacrimal gland may show areas of calcification without bony invasion. Extraocular muscle enlargement, soft tissue infiltration may also be evident.[1,2,7] I[1–3] labeled serum amyloid protein scintigraphy is a highly specific and sensitive diagnostic tracer for all types of amyloidosis.[4] Scintigraphy was not done in our case owing to lack of availability.

Differential diagnosis of lacrimal gland enlargement includes inflammatory disease, epithelial tumors and lymphomas. Dacryops and amyloid deposition are unusual causes of lacrimal gland enlargement. In patients such as ours who present with a progressive lacrimal gland enlarge ment with a homogenous mass on imaging, the chief differentials are lymphoproliferative disease, specific inflammatory conditions such as sarcoidosis, and epithelial tumors. These conditions are almost impossible to distinguish on imaging and biopsy is usually necessary to arrive at the correct diagnosis.[8]

Tissue biopsy in amyloidosis shows amorphous eosinophillic deposits with hematoxylin and eosin staining. With the Congo red stain, these deposits stain brick-red and show a green birefringence with polarized light. This feature is pathognomonic for amyloid deposits and is essential for diagnosis. There are different subtypes of amyloid such as light chain amyloid, amyloid-associated protein, β2 microglobulin and transthyretin amyloidodosis which are associated with different disease processes. Isolated amyloidosis is usually of light chain amyloid type. Immunohistochemistry can be used to distinguish between these subtypes. The diagnosis of systemic amyloidosis can be established by serum electrophoresis, rectal biopsy or abdominal subcutaneous fat biopsy.[1,2,4]

The management of patients with amyloidosis is complex and depends on associated systemic involvement. Treatment of choice in localized amyloidosis is surgical excision or mass debulking.[1,2,4] As illustrated by our case, lacrimal gland amyloidosis does not show any distinguishing clinical or imaging features and this underscores the importance of biopsy as a diagnostic technique in lacrimal gland masses.
